# Hints on Venesection in Chill of Intermittents

**Published:** 1837

**Authors:** 


Art. IV.—Hints on the Efficacy of Venesection in the Chill of Intermitent Fever. Extracted from a letter of Dr. T. Stith Malone, of Athens, Alabama.

Your Journal for January, 1837, contains some cases of intermittent fever, treated on Dr. MacIntosh's plan, of venesection in the cold stage; which reminded me of a few trials made in this quarter. In the year 1835, Dr. McDonald and myself, were in the poor-house of this county, (Limestone) and observed a pauper affected with intermittent fever, whom the chill was then coming over. I proposed bloodletting, to which he consented, as, to use his own language, his "head had nearly killed him during every shake." I immediately opened a vein, but could not extract more than 4 or 5 ounces. Dr. M. then performed the operation in his other arm, but with no better success, as to the quantity obtained. The chill, however, was immediately arrested, and a gentle glow of warmth, with moisture succeeded. He took a dose of cathartic pills, and had but another mild paroxysm.

In the next year, 1636, I treated two colored patients affected with intermittent fever, by venesection in the cold stage, followed by mild cathartics; And they both recovered, it seemed to me much more speedily, and without the lingering pains in the head and other parts of the body, which so frequently follow the routine practice of this country. Dr. M. has also pursued this practice with success in a number of cases. Both of us intend to follow it up, and will give you the results in due time.

---

We shall be happy to hear from Drs. Malone and McDonald on this interesting subject.—Ed.

